# Swimming ameliorates intervertebral disc degeneration accompanied by Rpl23a downregulation and changes in its immune-inflammatory pathway

**DOI:** 10.3389/fimmu.2026.1819987

**Published:** 2026-06-02

**Authors:** Yong Ji, Haixin Ma, Rong Tan, Xin Sha, Xin Li

**Affiliations:** 1 Department of Spine II, The Ninth Medical Center, Chinese People's Liberation Army (PLA) General Hospital, Beijing, China; 2 Department of Surgery II, Chinese People's Liberation Army General Hospital 63600 Hospital, Jiuquan, China; 3 Beijing Ditan Hospital, Capital Medical University, Beijing, China

**Keywords:** exercise intervention, fibroblast-nucleus pulposus crosstalk, immune microenvironment, immunometabolism, IVDD, NF-κB signaling, Rp23a

## Abstract

**Background:**

Intervertebral disc degeneration (IVDD) is characterized by persistent inflammation and extracellular matrix degradation, but the driving mechanism of immune dysregulation in the intervertebral disc remains unclear. Ribosomal protein L23a (Rpl23a) participates in various inflammatory diseases, but its role in the intervertebral disc immune microenvironment has not been studied. This study investigated the mechanism of Rpl23a in regulating immune interactions in IVDD and evaluated whether exercise intervention can interfere with this signaling axis.

**Methods:**

Bulk and single-cell RNA sequencing datasets (GSE189551, GSE153066) were analyzed to identify differentially expressed genes in IVDD and determine their cellular origins. Ligand-receptor interaction analysis was used to predict the immune cell communication network. *In vitro* experiments explored the role of Rpl23a in immune activation and NF-κB pathway activity. A rat IVDD model was established *in vivo* to evaluate the effect of swimming on Rpl23a-mediated inflammatory responses.

**Results:**

Rpl23a was significantly upregulated in degenerated intervertebral discs, mainly expressed in fibroblasts. Intercellular communication analysis revealed extensive crosstalk between Rpl23a-positive fibroblasts, macrophages, and T lymphocytes, contributing to the formation of a pro-inflammatory microenvironment. *In vitro* experiments showed that Rpl23a overexpression increased the secretion of TNF-α and IL-1β, activated NF-κB signaling, and further induced cell apoptosis and matrix catabolism; Rpl23a knockdown reversed these effects. Swimming alleviated IVDD in rats, accompanied by reduced Rpl23a expression, decreased immune cell infiltration, and inhibited NF-κB activation.

**Conclusion:**

Rpl23a is closely related to NF-κB signaling activation and may participate in the formation of a pathological immune microenvironment. Swimming can alleviate IVDD, accompanied by Rpl23a downregulation and NF-κB pathway inhibition, suggesting that Rpl23a may mediate the intervertebral disc protective effects of exercise and provide a theoretical reference for exercise therapy of IVDD.

## 1 Introduction

Low back pain and related dysfunctions are mostly attributed to IVDD, which is characterized by progressive structural and cellular damage of the intervertebral disc. Core manifestations of degenerated discs include reduced nucleus pulposus cells, imbalanced extracellular matrix homeostasis, narrowed intervertebral space, and increased inflammatory infiltration (1). Increasing evidence indicates that disordered immune-inflammatory microenvironment is a key driver of IVDD development. Among them, pro-inflammatory factors such as TNF-α and IL-1β accelerate cell apoptosis and matrix degradation through the NF-κB signaling axis (2–4). Despite progress in research on downstream effector mechanisms, the upstream molecular targets that initiate and sustain this inflammatory cascade remain unclear.

Ribosomal proteins were long thought to only participate in ribosome assembly and protein synthesis, but recent studies have confirmed that multiple ribosomal proteins exert non-ribosomal functions independent of protein synthesis, widely involved in cell proliferation, apoptosis, stress response, and inflammation regulation (5). Among them, ribosomal protein L23a (Rpl23a), an important multifunctional ribosomal protein, has been proven to regulate core inflammatory pathways such as NF-κB and MAPK through non-ribosomal mechanisms, playing a key regulatory role in rheumatoid arthritis, osteoarthritis, liver inflammation, and other inflammatory diseases (6) (7). Rpl23a can participate in the pathological process of chronic inflammatory diseases by regulating inflammatory factor secretion, immune cell activation, and matrix metabolic balance, suggesting that it may be a key molecule linking ribosomal dysfunction to local inflammation amplification. However, the expression, cellular localization, and biological function of Rpl23a in IVDD have not been reported, and whether it participates in the regulation of intervertebral disc immune microenvironment and can serve as an intervention target for IVDD remain unknown.

Exercise is a core non-pharmacological treatment for spinal diseases. Previous studies have confirmed that regular exercise can alleviate spinal degenerative lesions and improve patient function (8, 9). The beneficial mechanisms of moderate exercise include improving nutrient supply to avascular intervertebral discs, maintaining cellular metabolic balance, and reducing local inflammatory responses (10, 11). As a low-impact, full-body exercise, swimming is effective in delaying IVDD and promoting matrix synthesis (12, 13). Studies have reported that compared with high-load exercises such as running and resistance training, swimming does not exert excessive mechanical stress on intervertebral discs, has a lower injury risk, and is more suitable for long-term rehabilitation. Therefore, swimming is an ideal and clinically translatable exercise model for studying the protective mechanism of IVDD (13). Previous exercise intervention studies on IVDD have mostly focused on macroscopic mechanics, metabolism, and autophagy, and no key molecular switches targeting the immune microenvironment have been identified, especially lacking a complete mechanistic chain of “exercise → key molecule → immune-matrix crosstalk → intervertebral disc protection”. Therefore, whether Rpl23a mediates immune-inflammatory crosstalk in IVDD and whether exercise exerts protective effects by targeting this pathway remain unclear. Clarifying this issue will not only help identify the key molecular targets through which exercise protects intervertebral discs but also construct a novel exercise-responsive signaling axis for IVDD. As a potential upstream immune regulatory target, Rpl23a is expected to fill the mechanistic gap between current exercise interventions and immune microenvironment regulation, providing a new direction and scientific basis for precise rehabilitation and targeted drug development of IVDD. To fill the above research gaps, this study integrated bulk and single-cell transcriptome sequencing to screen IVDD-related genes, focused on analyzing the expression pattern of Rpl23a in different cell subsets and its function in regulating the immune-inflammatory microenvironment through an NF-κB-dependent mechanism, and evaluated whether swimming can exert protective effects by targeting this pathway. This study aims to deepen the understanding of IVDD pathogenesis and provide a molecular theoretical basis for exercise rehabilitation strategies.

## 2 Materials and methods

### 2.1 Intervertebral disc degeneration dataset

For this investigation, the publicly accessible single-cell RNA sequencing dataset GSE153066 was utilized. This dataset encompasses transcriptomic profiles of 35,846 cells derived from human intervertebral disc tissue, enabling high-resolution exploration of cellular heterogeneity and molecular signatures associated with IVDD.

### 2.2 Single-cell data preprocessing and quality control

Computational analyses were executed within the R software environment, with the Seurat package (v4.1.1) serving as the primary analytical tool. Given the substantial data volume, computational parameters were configured with a 32 GB memory threshold, and single-threaded processing was implemented to maintain stability. A batch processing strategy optimized for memory efficiency was employed. Raw sequencing data underwent conversion to sparse matrix format prior to integration into a comprehensive gene-by-cell expression matrix.

Quality control procedures commenced with Seurat object initialization applying dual criteria: genes detectable in minimum three cells and cells expressing at least 200 genes. For each cellular entity, four quality metrics were computed: (1) proportion of mitochondrial transcripts, (2) proportion of ribosomal gene transcripts, (3) total gene count, and (4) total UMI count. Through graphical evaluation employing violin and scatter plots, rigorous filtering parameters were established. Cells exhibiting gene counts between 300 and 4,000 were retained, while those with mitochondrial transcript proportions exceeding 10%—indicative of potential cellular damage or death—were excluded. This filtration strategy preserved approximately 90% of the original cellular population for subsequent analytical steps.

### 2.3 Data normalization, feature selection, and dimensionality reduction

Normalization of gene expression values across cells was accomplished using the LogNormalize method with a scale factor of 10,000. Identification of highly variable genes was performed via the variance stabilizing transformation (vst) algorithm, selecting the top 2,000 features that optimally capture distinct biological states.

Principal Component Analysis (PCA) was subsequently conducted on the selected highly variable gene set. Examination of the elbow plot, which visualizes the contribution of individual principal components to overall data variance, guided the selection of the initial 15 principal components for downstream applications, as these effectively encapsulated predominant biological variation.

### 2.4 Cell clustering, visualization, and annotation

Using the designated 15 principal components, the FindNeighbors function facilitated construction of a K-Nearest Neighbor (KNN) graph. Unsupervised clustering was accomplished through application of the Louvain algorithm via the FindClusters function. Systematic evaluation of multiple resolution parameters (0.2, 0.5, 0.8) identified 0.5 as yielding the most biologically interpretable cluster configurations. Dimensionality reduction for visualization employed the UMAP algorithm to project high-dimensional data into two-dimensional space, revealing relationships between identified cell clusters.

For each cluster, marker genes exhibiting specific high expression were identified using the FindAllMarkers function with Wilcoxon rank-sum test under default parameters. Selection criteria required gene expression in minimum 25% of cells within the cluster and average log2 fold change ≥ 0.25. The top five marker genes per cluster were extracted for annotation purposes. Drawing upon published intervertebral disc cell atlas literature, a tissue-specific marker gene panel was assembled encompassing nucleus pulposus cells, annulus fibrosus cells, cartilage endplate cells, stem/progenitor cells, immune populations, and endothelial cells. The AddModuleScore function computed enrichment scores for each cell across marker gene sets, with cell type assignment based on highest enrichment score exceeding the 0.1 threshold. Cells failing to meet this criterion were designated as “Unknown.”

### 2.5 Cross-cluster expression analysis of core genes

To corroborate findings from preceding PPI network analyses, systematic localization of identified core genes was undertaken. A multi-strategy matching pipeline was developed to reconcile nomenclature discrepancies across databases—including variations in letter case, symbol formatting, and version designations—ensuring accurate gene identification within the current dataset.

For successfully matched core genes, spatial expression patterns across the complete cellular landscape were visualized through UMAP feature plots. Quantitative comparisons of expression levels across annotated cell types were performed using violin and dot plots. Detailed characterization of Rpl23a expression across cellular subpopulations was conducted as a representative example.

### 2.6 Cell-cell communication analysis

Intercellular communication networks were investigated using the CellChat analytical tool, which leverages ligand-receptor interaction databases to systematically infer and compare communication patterns between pathological (degenerated) and healthy conditions across diverse cell types. This approach facilitates identification of potentially aberrant intercellular signaling mechanisms contributing to IVDD pathogenesis.

### 2.7 Cell culture and genetic manipulation

Human nucleus pulposus cells (NPCs, Procell, Cat. No. CP-H097) were routinely cultured in complete medium (Procell, Cat. No. CM-H097) at 37 °C with 5% CO_2_. The experimental groups were set as follows:

- Control group: normal untransfected nucleus pulposus cells.
- shNC group: cells transfected with shRNA negative control virus.
- shRpl23a group: cells transfected with Rpl23a silencing virus.
- oeNC group: cells transfected with empty vector overexpression virus.
- oeRpl23a group: cells transfected with Rpl23a overexpression virus.

Transfection efficiency was detected by RT-qPCR and Western blot 48 hours after transfection. Experimental groups: control group, sh-NC group, sh-Rpl23a group, oe-NC group, oe-Rpl23a group, with 6 replicate wells per group.

### 2.8 Cell viability assay

The CCK-8 kit (Dojindo, Japan) was used to detect the effect of Rpl23a on nucleus pulposus cell viability. Cells were seeded in 96-well plates at 5×10³ cells/well, 10 μL of CCK-8 solution was added to each well 48 hours after transfection, incubated at 37 °C for 2 hours, and absorbance at 450 nm was detected using a microplate reader (BioTek, USA). Six replicate wells were set per group, and the experiment was independently repeated 3 times.

### 2.9 Animal model establishment and exercise intervention

Thirty 6-week-old healthy male Sprague-Dawley (SD) rats (weighing 180–220 g, Beijing Vital River Laboratory Animal Technology Co., Ltd.) were randomly divided into sham operation group, model group, and exercise intervention group, with 10 rats in each group. Before modeling, anesthesia was induced with 3.0% isoflurane (100% oxygen, flow rate 2 L/min) and maintained with 1.5%–2.0% isoflurane during surgery. Anesthesia depth was monitored by corneal reflex, paw withdrawal reflex, and respiratory rate, and the concentration was adjusted timely. The L4-L5 IVDD model was constructed by percutaneous intervertebral disc puncture with a 21G needle: after anesthesia, the target intervertebral disc was exposed, the 21G injection needle was vertically inserted into the nucleus pulposus and pulled out after staying for 10 seconds; the sham operation group only exposed the intervertebral disc without puncture.

The exercise intervention group started swimming training on the 7th day after surgery, 5 times a week, 30 minutes each time, for a total of 8 weeks. The size of the swimming box was 100×60×60 cm, and the water temperature was 32 ± 2 °C. Within 24 hours after the last intervention, rats were humanely euthanized: deeply anesthetized with 5.0% isoflurane (100% oxygen, flow rate 2 L/min) until reflexes and spontaneous breathing completely disappeared, followed by cervical dislocation. Immediately after confirmation of death, L4-L5 intervertebral disc tissues were separated and collected for subsequent detection.

### 2.10 detection indicators and methods

#### 2.10.1 RT-qPCR detection

Total RNA from cells and tissues was extracted using TRIzol reagent (Invitrogen, 15596026) and reverse-transcribed into cDNA. The SYBR Green method (Servicebio, G3345-100) was used to detect the mRNA expression levels of Rpl23a, TNF-α, IL-1β, IL-6, type II collagen, aggrecan, and MMP-13. Primer sequences (Beijing Tsingke Biotechnology Co., Ltd.) are shown in **Table 1** and **Table 2**. GAPDH was used as an internal reference, and the relative expression was calculated using the 2^-^^Δ^Δ^Ct method.

**Table 1:** Primer sequences for *in vitro* RT-qPCR.

Name	Primer sequence	Product length	Tm	Length	GC%	Self complementarity	Self 3’ complementarity
Rpl23a	5’-GACAGCCCAAATATCCTCGG-3’	234	58.12	20	55.00	4.00	2.00
	3’-TCTCTCCATCAGGCCGAATC-5’		58.96	20	55.00	4.00	3.00
TNF-α	5’-AGCCCATGTTGTAGCAAACC-3’	100	58.74	20	55.00	4.00	0.00
	3’-GTTATCTCTCAGCTCCACGC-5’		58.15	20	55.00	4.00	2.00
IL-1β	5’-TCTTCATTGCTCAAGTGTCTGA-3’	120	57.66	22	40.91	4.00	3.00
	3’-GTTTAGGGCCATCAGCTTCA-5’		57.87	20	50.00	4.00	1.00
IL-6	5’-CCACTCACCTCTTCAGAACG-3’	202	57.93	20	55.00	5.00	3.00
	3’-CCAGGCAAGTCTCCTCATTG -5’		58.25	20	55.00	3.00	3.00
Collagen II	5’-GAGCCATGATTCGCCTCG-3’	101	58.36	18	61.11	4.00	2.00
	3’-GCCAGCCTCCTGGACAT-5’		58.56	17	64.71	5.00	2.00
Aggrecan	5’-ATGGACACCCCATGCAATTT-3’	432	58.04	20	45	5.00	3.00
	3’-CTGGAAGCTCTTCTCAGTGG -5’		57.62	20	55	8.00	1.00
MMP-13	5’-TCCAGTCTCTCTATGGTCCAG-3’	225	57.70	21	52.38	5.00	3.00
	3’-CAGCATCAATACGGTTGGGA-5’		57.96	20	50.00	3.00	1.00
GAPDH	5’-AATGAATGGGCAGCCGTTAG-3’	662	58.61	20	50.00	5.00	0.00
	3’-CATGGACTGTGGTCATGAGT-5’		57.22	20	50.00	600	2.00

**Table 2:** Primer sequences for *in vivo* qRT-PCR.

Name	Primer sequence	Product length	Tm	Length	GC%	Self complementarity	Self 3’ complementarity
Rpl23a	5’-CAATACTCTGATACGGCCTGA-3’	193	56.99	21	47.62	4.00	2.00
	3’-TAAGGTAAAGCGAGCTGGGA -5’		58.44	20	50.00	4.00	0.00
Collagen II	5’-TGCAGAATGGGCAGAGGTAT-3’	202	58.78	20	50.00	4.00	2.00
	3’-TTTGGCCCTAATTTTCCACTG-5’		56.67	21	42.86	5.00	3.00
Aggrecan	5’-ACCTGTGTGAGATCGACCA-3’	208	57.95	19	52.63	4.00	2.00
	3’-TTCAGACCGATCCACTGGTA-5’		57.77	20	50.00	5.00	3.00
GAPDH	5’-CCCTCAACAGGGATGCTTAC -3’	101	57.96	20	55.00	4.00	2.00
	3’-GATACGGCCAAATCCGTTCA-5’		58.34	20	50.00	6.00	1.00

#### 2.10.2 Western blot analysis

Total protein was extracted using RIPA lysis buffer (Beyotime, China), separated by SDS-PAGE electrophoresis, transferred to a membrane, and blocked. Primary antibodies were incubated overnight at 4 °C: Rpl23a (Proteintech, 16386-1-AP), phosphorylated p-NF-κB p65 (Beyotime, AF5875), p-NF-κB p65 (Proteintech, 80979-1-RR), Bax (Proteintech, 50599-2-Ig), Bcl-2 (Beyotime, AG1225), and GAPDH (Proteintech, 81640-5-RR). Then, HRP-labeled secondary antibody (1:5000) was incubated for 1 hour, and ECL luminescence detection (BeyoECL Plus, Beyotime) was performed. Gray value analysis was performed using ImageJ software, normalized with GAPDH as an internal reference.

#### 2.10.3 ELISA detection

The supernatant of intervertebral disc tissue homogenate was taken, and the protein concentrations of Rpl23a (Wuhan Cloud-Clone Science and Technology Co., Ltd., USEP643Mi), TNF-α (Solarbio, SEKR-0009), IL-1β (Solarbio, SEKR-0002), IL-6 (Solarbio, SEKR-0005), and MMP-13 (mlbio, ml002962) were detected strictly in accordance with the instructions of the rat-specific ELISA kits.

#### 2.10.4 General status and behavioral tests

##### 2.10.4.1 Body weight monitoring

The body weight of rats was weighed with an electronic scale at the same time every week, and the body weight change trend of each group was recorded.

##### 2.10.4.2 Mechanical withdrawal threshold

The mid−plantar region of the rat hind paw was stimulated with von Frey filaments. The minimum filament force that triggered rapid paw withdrawal, leg lifting, or paw licking was recorded. Each test was repeated 3 times, and the average value was calculated to reflect the degree of mechanical hyperalgesia.

##### 2.10.4.3 Thermal withdrawal latency

Rats were placed on a hot plate apparatus with the temperature adjusted to (55.0 ± 0.5)°C. The latency from paw contact with the hot plate to paw licking or lifting was recorded. Each test was repeated 3 times (at 10−minute intervals), and the average value was calculated to reflect the degree of thermal hyperalgesia.

### 2.11 Statistical analysis

Data were processed using SPSS 22.0 software, and measurement data were expressed as mean ± standard deviation (x¯ ± s). One-way analysis of variance (ANOVA) was used for comparison between multiple groups; LSD-t test was used for pairwise comparison when variance was homogeneous, and Tamhane’s T2 test was used when variance was heterogeneous. P<0.05 was considered statistically significant.

## 3 Results

### 3.1 Data quality control and principal component analysis

**Figure 1:**
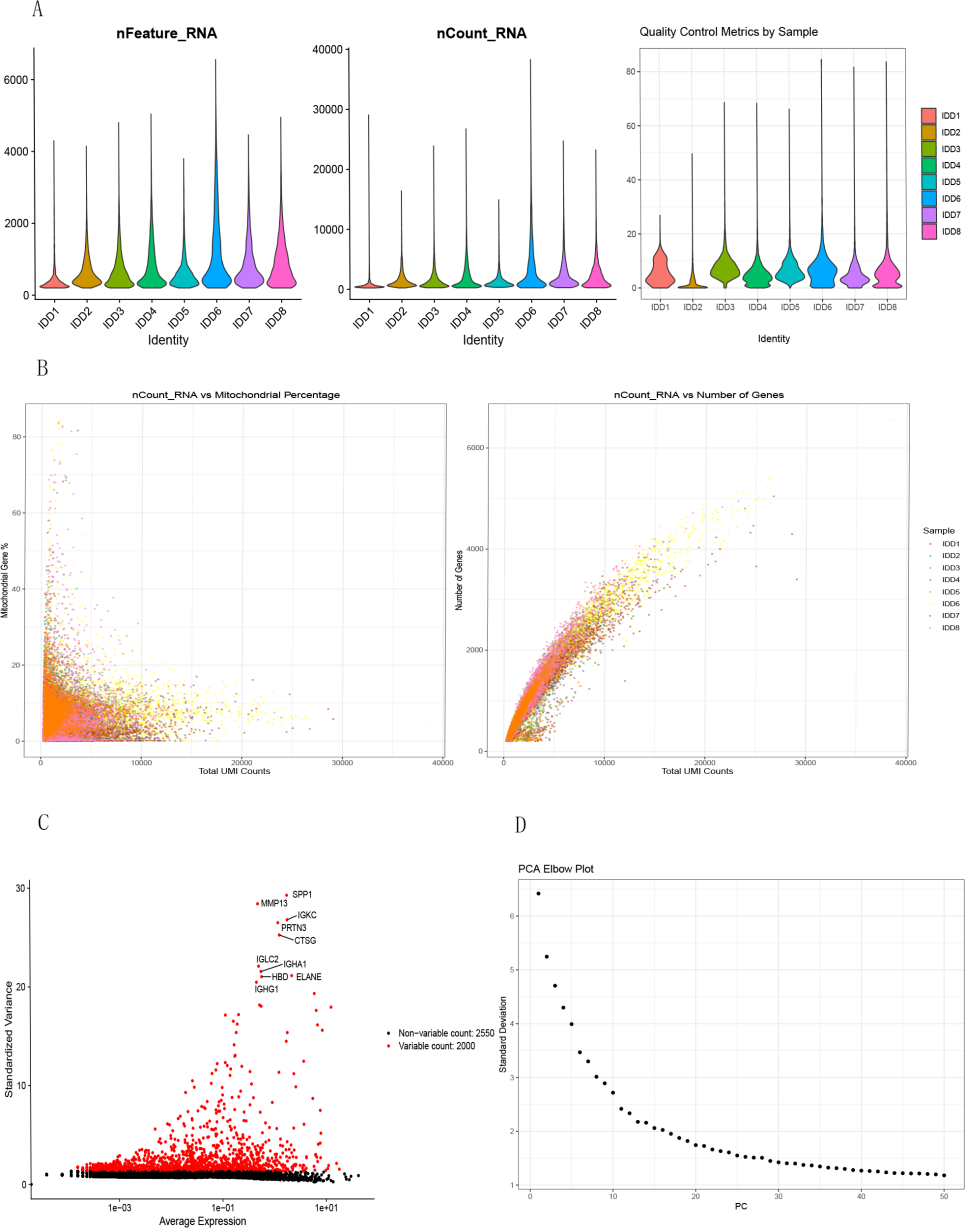
Preprocessing and quality control analysis results of single-cell RNA sequencing data from intervertebral disc tissue. **(A)** Distribution of the number of genes detected (nFeature_RNA), total UMI counts (nCount_RNA), and quality control metrics across different samples. **(B)** Scatter plot showing the relationship between total UMI counts and the percentage of mitochondrial genes (left), and between total UMI counts and the number of detected genes (right). **(C)** Scatter plot of gene mean expression versus standard deviation (highlighting highly variable genes). **(D)** Elbow plot from Principal Component Analysis (PCA).

A single-cell RNA sequencing dataset comprising 27,504 genes (corresponding to sample IDs IDD1-IDD8) was analyzed using the “Seurat” package. Through a detailed review of gene features and sequencing depth for each sample, it was observed that nCount_RNA ranged from 0 to 40,000, nFeature_RNA was mainly distributed between 20 and 80, and percent_mt (the percentage of mitochondrial genes) ranged from 0% to 60% (**Figure 1A**). Consequently, cell quality control measures were implemented (nFeature_RNA > 40, nCount_RNA > 10,000, percent_mt < 20%) to filter out low-quality samples and technical noise.

Principal Component Analysis (PCA) revealed the number of core principal components within this dataset. Evaluation using the `ElbowPlot` function showed that the standard deviation of the principal components plateaued after a specific inflection point. This inflection point corresponds to the Xth principal component (where X is determined based on the actual inflection point in the elbow plot, e.g., between 10 and 20), specifically PC9~PC19 represented the inflection points of the standard deviation curve (**Figure 1D**). Employing a significance threshold of P < 0.05 for statistical analysis, the study found that the first X principal components exhibited significant differences and were worthy of inclusion in further analysis.

Results from the highly variable gene screening showed that 2,000 highly variable genes were identified from the total of 27,504 genes (Variable count: 2,000), while the total number of non-variable genes was 25,504 (Non-variable count: 25,504). Genes such as SPP1, MMP13, IGKC, and PRTN3 demonstrated exceptionally high expression variability, serving as core markers for inter-sample differences (**Figure 1C**). Further validation via scatter plots revealed a positive correlation with a marginally diminishing trend between nCount_RNA and nFeature_RNA, indicating a match between sequencing depth and gene capture efficiency. No significant correlation was observed between nCount_RNA and percent_mt, which aligns with the indicator correlation characteristics of high-quality samples (**Figure 1B**). The preliminary quality control results demonstrated that the distribution of nCount_RNA and nFeature_RNA for most samples was concentrated and reasonable, with sequencing depth and gene diversity meeting the requirements for downstream analysis (**Figure 1A**).

### 3.2 UMAP clustering analysis and identification of cell types and marker genes

**Figure 2:**
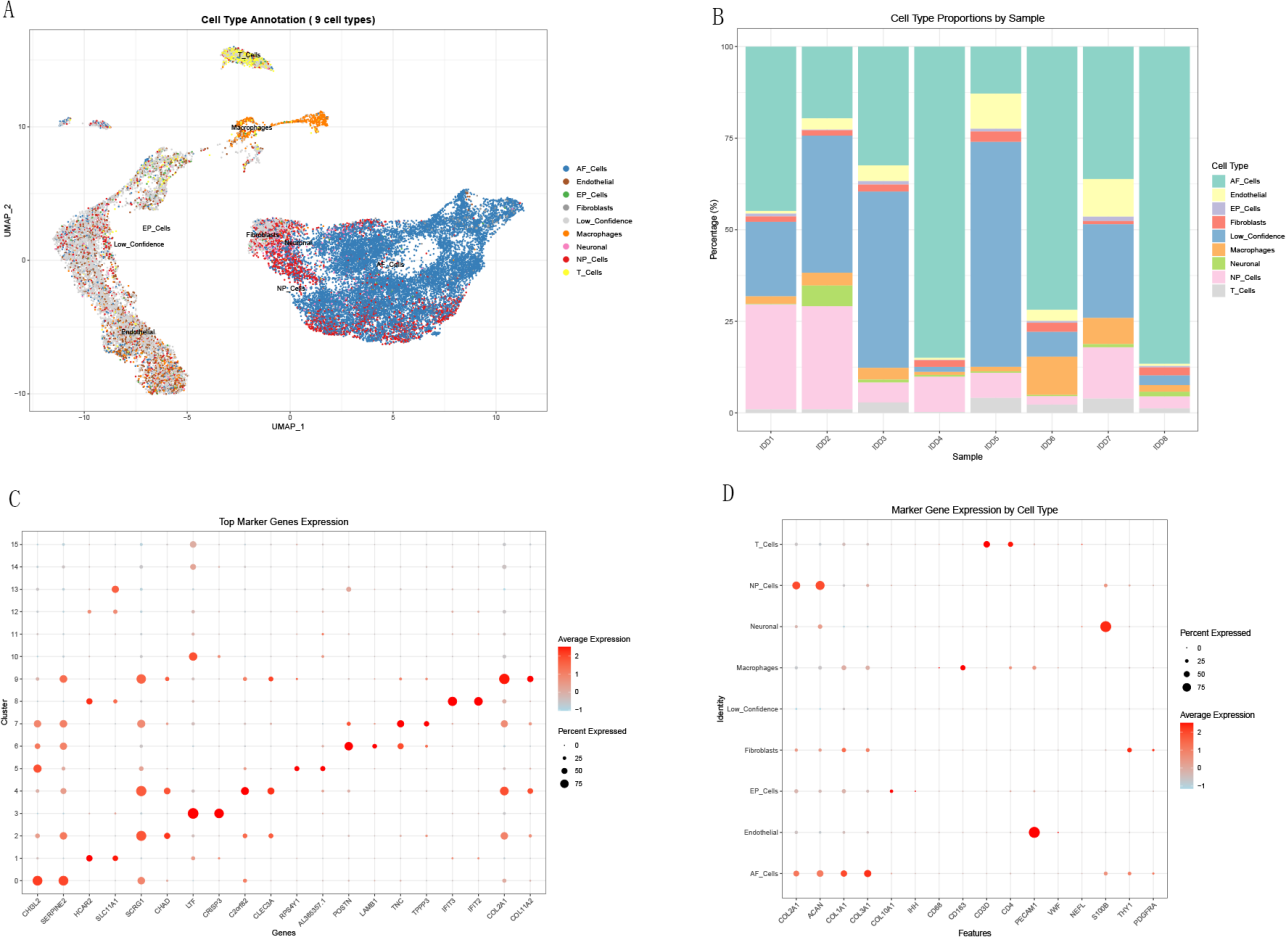
Results of cell clustering and annotation analysis for single-cell RNA sequencing data of intervertebral disc tissue. **(A)** UMAP dimensionality reduction visualization plot showing the distribution of different cell types in two-dimensional space. **(B)** Stacked bar chart illustrating the proportion of different cell types across each sample. **(C)** Heatmap displaying the expression of marker genes for each cell cluster. **(D)** Feature plots showing the expression characteristics of marker genes across different cell types.

Following standardized correction of the single-cell data from eight samples (IDD1-IDD8) using bioinformatics analysis tools, a two-dimensional visualization space was constructed via the UMAP dimensionality reduction algorithm. All cells were clearly clustered into multiple distinct clusters. Based on the expression characteristics of marker genes and functional annotations, these clusters were identified as nine distinct cell types: AF_Cells, Endothelial Cells, EP_Cells, Fibroblasts, Low_Confidence Cells, Macrophages, Neuronal Cells, NP_Cells, and T_Cells (**Figure 2A**). Analysis of cell type proportions revealed that Fibroblasts and Macrophages were the predominant cell types across all samples, followed by AF_Cells and NP_Cells. In contrast, Endothelial Cells, T_Cells, and Neuronal Cells constituted relatively lower proportions. Low_Confidence Cells were present at minimal levels across all samples, indicating high reliability of the cell typing results (**Figure 2B**). Further analysis of cell type-specific marker genes identified characteristic markers for each type: Macrophages were marked by high expression of CHI3L2, SERPINE2, and HCAR2; Fibroblasts by COL1A1, COL3A1, THY1, and PDGFRA; NP_Cells by COL2A1, ACAN, and IHH; T_Cells by CD3D and CD4; and Endothelial Cells by VWF and PECAM1. These marker genes consistently exhibited both high expression levels and high positivity rates within their respective cell types, confirming their utility as specific diagnostic markers for cell type identification (**Figures 2C, D**).

### 3.3 Analysis of cross-cluster expression results for core genes

To validate the results from the previous PPI network analysis, we systematically localized the screened core genes. We developed a multi-strategy matching pipeline to address inconsistencies in gene nomenclature across different databases (e.g., letter case, symbol format, version numbers), ensuring that the core gene list could be accurately identified within the current dataset. Ultimately, 11 out of 14 candidate core genes were successfully matched and detected, with no genes showing obvious low or negative expression, establishing a reliable foundation for subsequent analysis.

**Figure 3:**
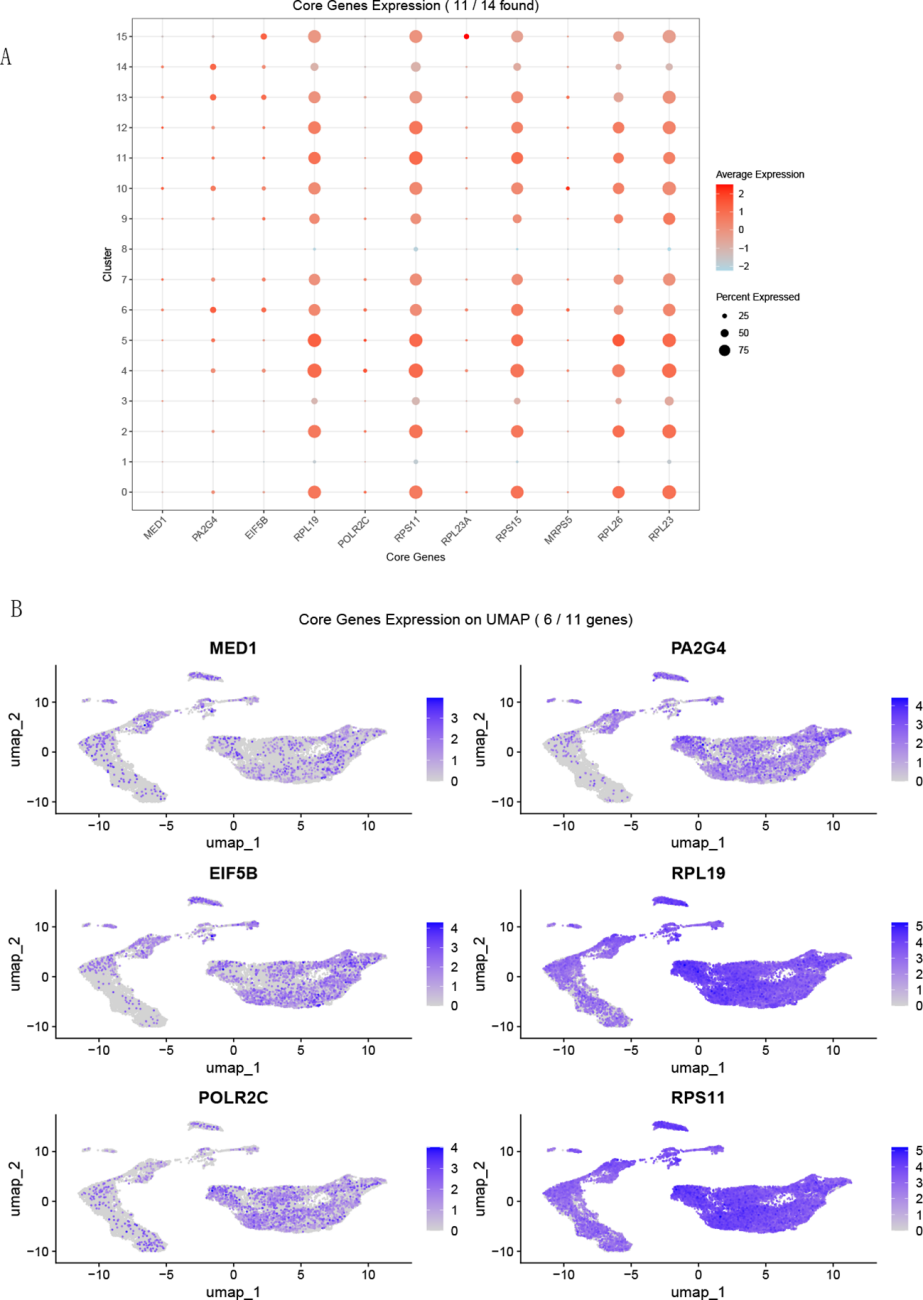
Analysis of expression characteristics of IDD core genes in the single-cell population of intervertebral discs. **(A)** Heatmap showing the expression of core genes across different cell clusters. **(B)** Feature plot illustrating the expression distribution of core genes in UMAP space.

For the successfully matched core genes, we visualized their spatial expression patterns across the entire cell population using UMAP feature plots and quantitatively compared their expression differences across annotated cell types using violin plots and dot plots. Firstly, analysis of UMAP feature plots for six key core genes (PA2G4, MED1, RPL19, EIF5B, POLR2C, RPS11) revealed that the high-expression signals for all core genes were predominantly and distinctly concentrated within the independent cluster corresponding to Fibroblasts in the UMAP space, with clear boundaries and no widespread dispersed expression. This clearly confirms the significant cell type specificity of the core genes (**Figure 3B**). Dot plots further quantifying the core genes showed that five genes, including PA2G4, EIF5B, and RPL19, exhibited high expression abundance (average expression level 11~14), while six genes, including MED1 and RPL23A, showed moderate expression abundance (average expression level 6~9). Moreover, seven genes had an expression positivity rate ≥50%, indicating broad coverage and verifying the stable high-expression characteristics of the core genes within the target cell population (**Figure 3A**).

**Figure 4:**
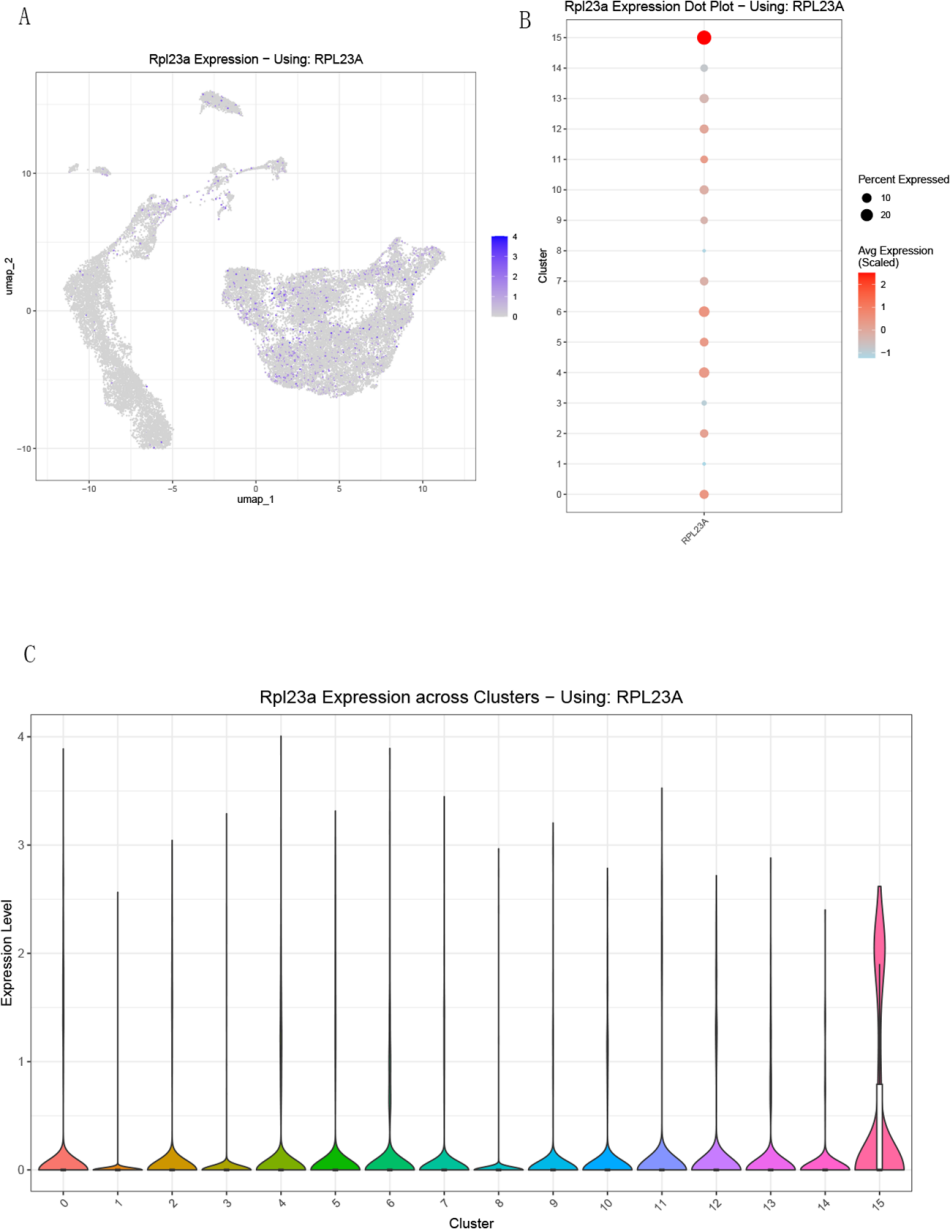
Analysis of expression characteristics of the Rpl23a gene in the single-cell population of intervertebral discs. **(A)** Feature plot showing the expression distribution of Rpl23a in UMAP space. **(B)** Dot plot illustrating the expression of Rpl23a across different cell clusters. **(C)** Violin plot depicting the expression levels of Rpl23a in different cell clusters.

This study specifically used the Rpl23a gene as an example to detail its specific expression characteristics across various cell subpopulations. The UMAP feature plot clearly showed that the high-expression signal of Rpl23a was precisely enriched within the Fibroblast cluster, with no abnormal outlier expression points (**Figure 4A**). A cross-cluster violin plot further confirmed that the median expression level of Rpl23a in Fibroblasts was approximately 2.5, significantly higher than in other cell types (expression levels only 0~1), and its expression distribution was concentrated with no extreme outliers (**Figure 4C**). A dedicated dot plot quantifying Rpl23a validated its average expression level at approximately 9 and a positivity rate of 50%, consistent with the overall expression pattern of the core genes, fully supporting its reliability as a Fibroblast-specific core gene (**Figure 4B**).

### 3.4 Analysis of intercellular communication networks and core molecular regulatory mechanisms

**Figure 5:**
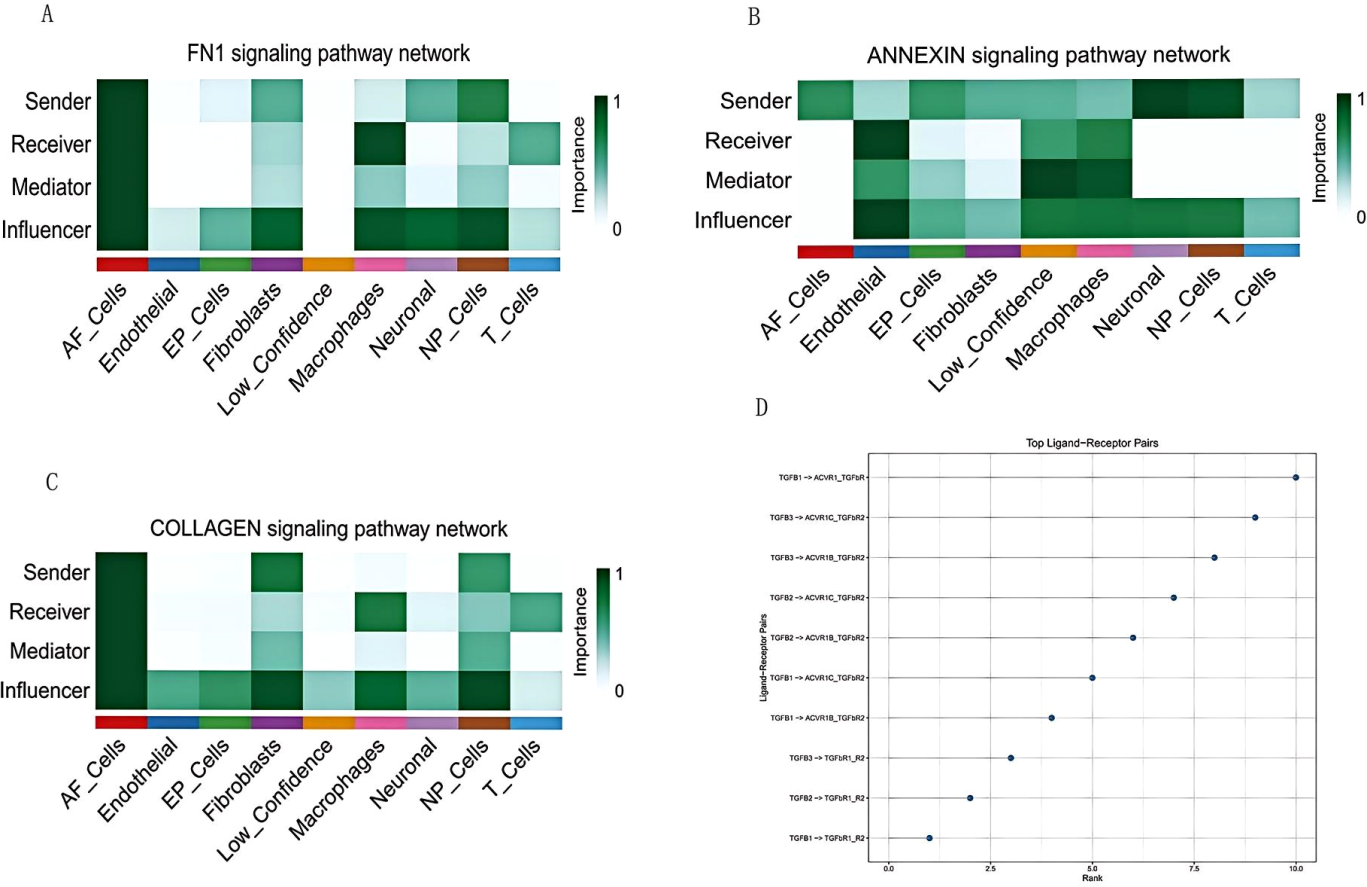
Analysis results of intercellular communication networks in intervertebral disc tissues under different conditions. **(A)** Heatmap of intercellular communication counts in intervertebral disc tissues. **(B)** Heatmap of intercellular communication weights in intervertebral disc tissues. **(C)** Heatmap of interactions between intercellular signaling pathways. **(D)** Patterns of incoming and outgoing signaling pathways across different cell types.

**Figure 6:**
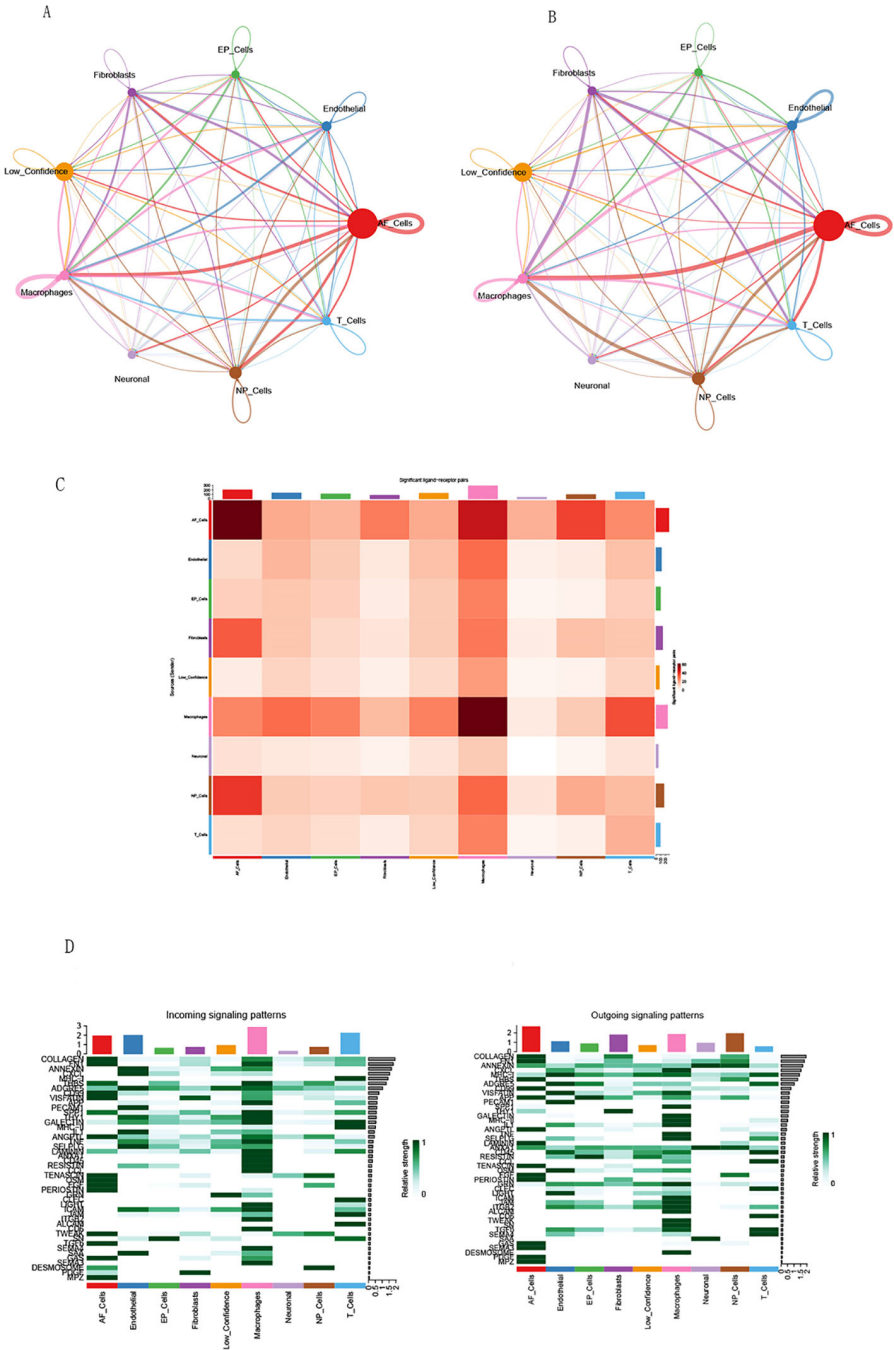
Analysis results of intercellular regulatory networks and ligand-receptor pairs for key signaling pathways in intervertebral disc tissue. **(A)** Heatmap of the intercellular regulatory network for the FN1 signaling pathway. **(B)** Heatmap of the intercellular regulatory network for the ANNEXIN signaling pathway. **(C)** Heatmap of the intercellular regulatory network for the COLLAGEN signaling pathway. **(D)** Ranking scatter plot of core ligand-receptor pairs.

To elucidate the molecular mechanisms of coordinated cellular regulation within the samples, a systematic analysis of communication features among the nine cell types was conducted using the CellChat tool. The intercellular communication count heatmap revealed that Fibroblasts and Macrophages form a bidirectional, strong communication core, with their interaction frequency significantly higher than other cell pairs. In contrast, communication frequencies involving Low_Confidence cells, Endothelial cells, and T_Cells were extremely low (**Figure 5A**). The communication weight heatmap further pinpointed functionally critical interactions. Bidirectional communication between Fibroblasts and Macrophages exhibited the highest weight, with the “Macrophages → Fibroblasts” direction showing a significantly greater weight than the reverse, establishing it as the dominant direction of the core regulatory axis. Fibroblasts → NP_Cells was identified as an important auxiliary communication link (**Figure 5B**). The significant ligand-receptor (LR) pair heatmap validated the molecular specificity of the core communication axis. The “Macrophages → Fibroblasts” direction was enriched with a large number of statistically significant LR pairs, far exceeding those of other cell pairs, confirming the reliability of molecular communication in this direction (**Figure 5C**). Signal reception pattern analysis indicated that Fibroblasts, acting as the primary signal-integrating cells, were significantly enriched for repair-related pathway signals such as TGFβ, PDGF, and IL6, with the highest relative intensity, while Macrophages primarily received anti-inflammatory feedback signals (**Figure 5D**). Outgoing signal pattern clustering analysis demonstrated that intercellular signal transmission could be categorized into two stable clustering patterns (Cophenetic coefficient ≈ 0.99, Silhouette coefficient ≈ 1.00), with clear differentiation between the signal-sending characteristics of core and auxiliary cells (**Figure 5D**). Analysis of specific signaling pathways revealed that the COLLAGEN, FN1, and ANNEXIN pathways all centered on the Fibroblasts-Macrophages axis, focusing on tissue remodeling, matrix synthesis initiation, and inflammation-repair coupling, respectively (**Figures 6A–C**). The Top 10 significant LR pairs further pinpointed core molecular targets, all originating from the TGFβ signaling pathway. Ligands included TGFB1, TGFB2, and TGFB3, with receptors such as ACVR1_TGFbR and TGFbR1_R2 family complexes. Among these, TGFB1→ACVR1_TGFbR and TGFB1→TGFbR1_R2 were identified as the highest-ranked key interaction pairs (**Figure 6D**).

In summary, a hierarchical communication network was formed in the sample: with “macrophages → fibroblasts” as the core regulatory axis and core LR pairs of the TGFβ pathway as molecular switches, synergizing with collagen/FN1/annexin functional pathway clusters, dominating the “inflammatory response-tissue repair” process through a closed-loop regulation of “signal initiation-integration-execution-feedback”.

Based on the above bioinformatics findings, we further conducted *in vitro* and *in vivo* experiments to explore the specific mechanism of Rpl23a and the effect of exercise intervention.

### 3.5 Rpl23a regulates nucleus pulposus cell viability, inflammatory response, and matrix metabolism

#### 3.5.1 Rpl23a inhibits nucleus pulposus cell viability

**Figure 7:**
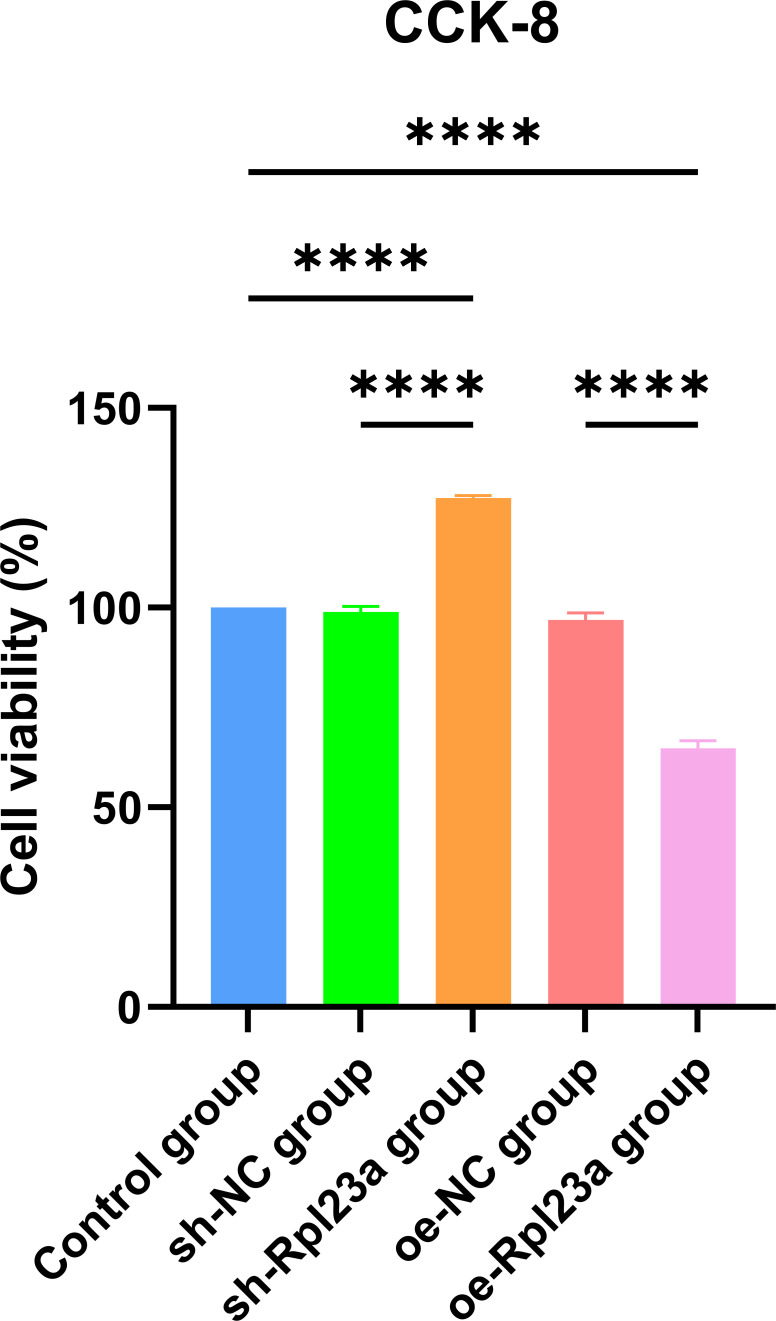
Rpl23a regulates nucleus pulposus cell viability. Cell viability was detected by CCK-8–48 hours after transfection, grouped as Control, shNC, shRpl23a, oeNC, oeRpl23a. *P<0.05, **P<0.01, ***P<0.001, ****P<0.0001. Data are expressed as mean ± standard deviation (n=3).

CCK-8 assay results showed (**Figure 7**) that compared to the control group, silencing Rpl23a (sh-Rpl23a group) significantly increased nucleus pulposus cell viability at 48h and 72h post-transfection (P < 0.05). Conversely, overexpressing Rpl23a (oe-Rpl23a group) significantly inhibited cell viability (P < 0.01). This suggests that high expression of Rpl23a may impair the survival capacity of nucleus pulposus cells.

#### 3.5.2 Rpl23a promotes inflammatory factor expression and inhibits matrix synthesis

**Figure 8:**
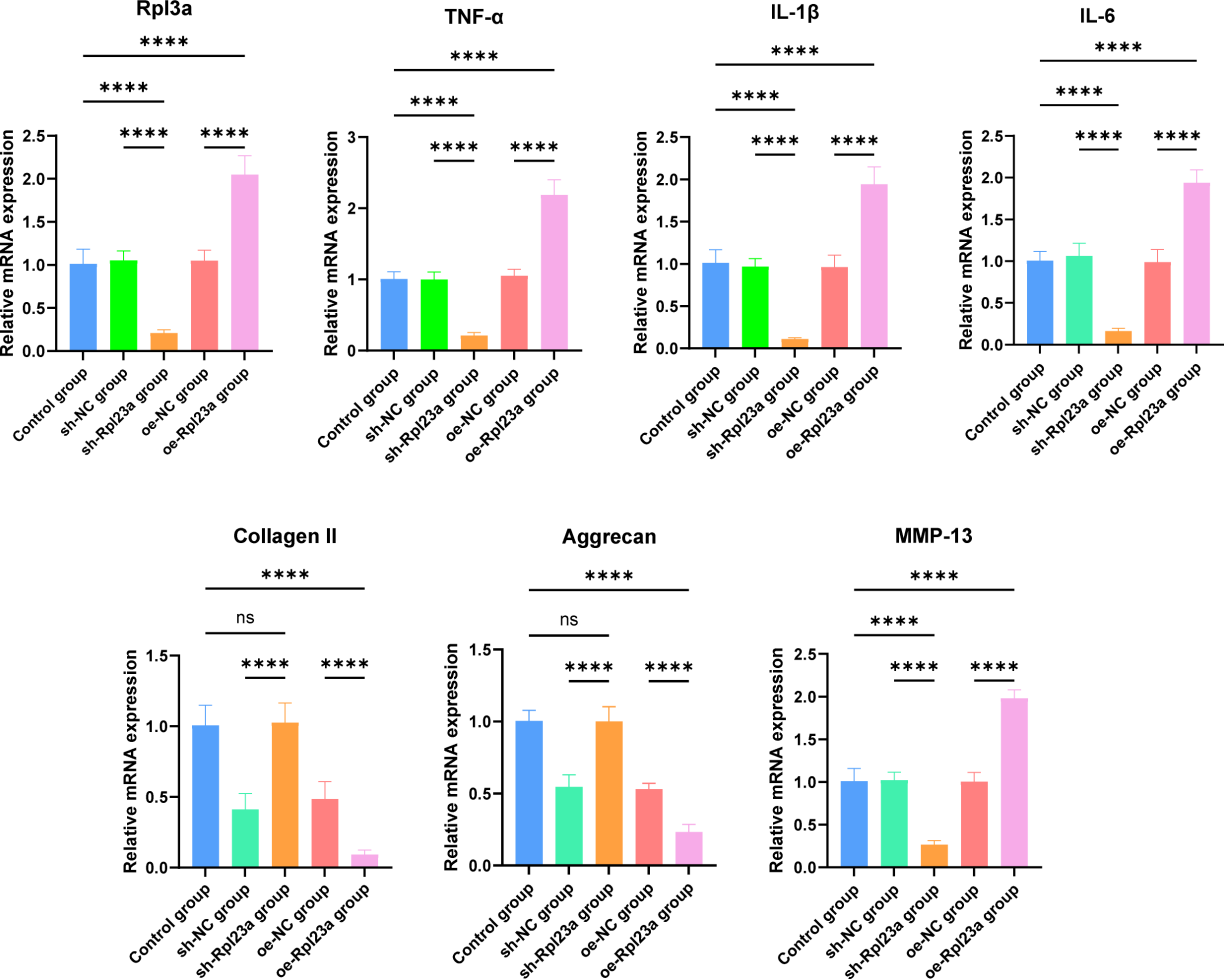
Rpl23a modulates the expression of inflammatory and matrix-related genes in nucleus pulposus cells. RT-qPCR was used to detect the mRNA levels of Rpl23a, TNF-α, IL-1β, IL-6, MMP-13, type II collagen, and aggrecan, with GAPDH as an internal reference. *P<0.05, **P<0.01, ***P<0.001, ****P<0.0001. Data are expressed as mean ± standard deviation (n=6), data normalization: normalized to the Control group using the 2^(-ΔΔCt) method with GAPDH as an internal reference.

RT-qPCR results (**Figure 8**) showed that in the sh-Rpl23a group, mRNA expression of pro-inflammatory factors (TNF-α, IL-1β, IL-6) and the matrix-degrading enzyme MMP-13 was significantly downregulated (P < 0.05), while expression of matrix synthesis genes Collagen II and Aggrecan was significantly upregulated (P < 0.01). The oe-Rpl23a group showed the completely opposite trend. Rpl23a’s own mRNA expression was also effectively modulated in the corresponding groups, confirming transfection efficiency.

#### 3.5.3 Rpl23a promotes cell apoptosis by activating the NF-κB pathway

**Figure 9:**
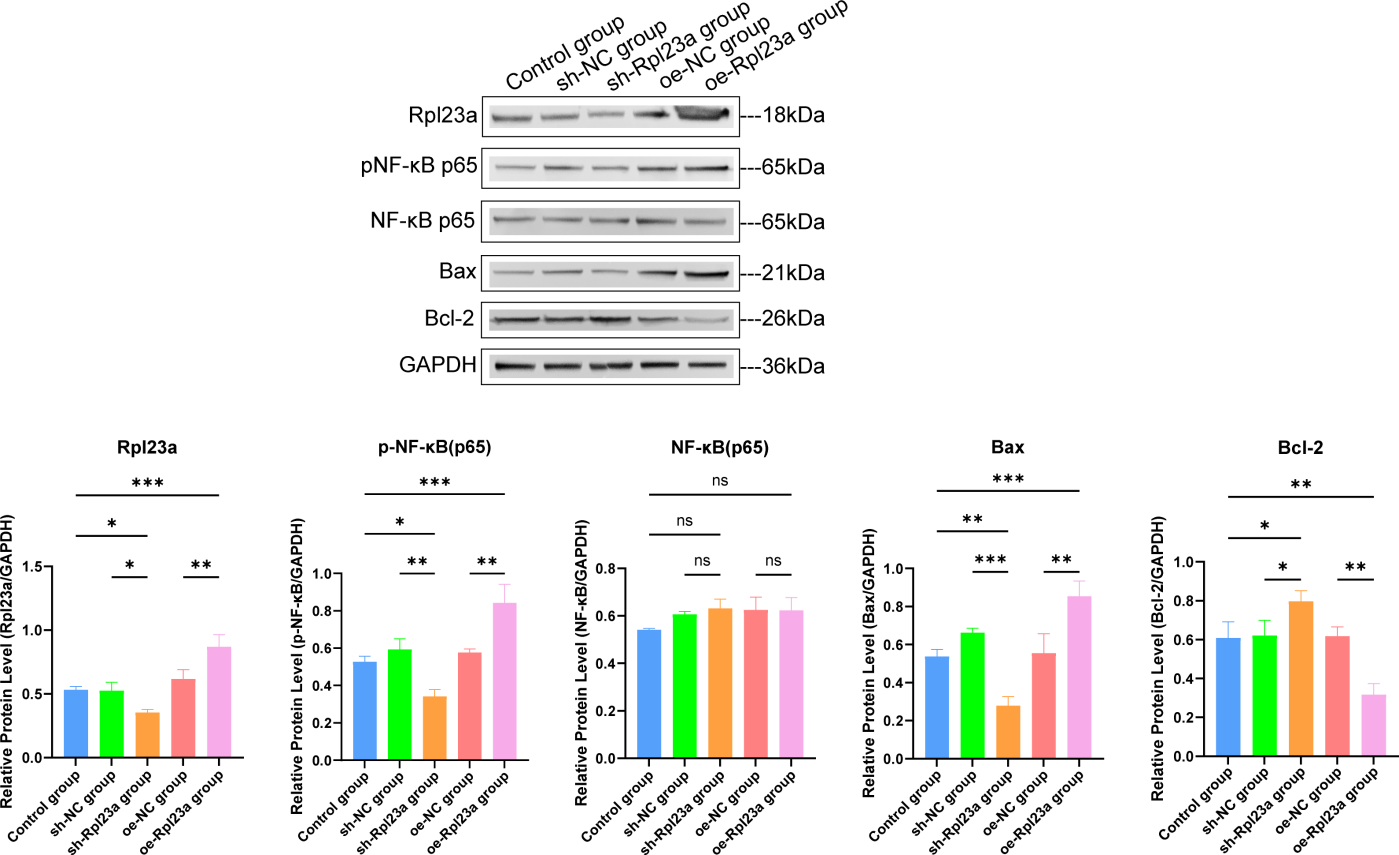
Rpl23a influences NF-κB pathway activation and apoptosis-related protein expression. Representative Western blot images of Rpl23a, p-NF-κB p65, total NF-κB p65, Bax, Bcl-2, and GAPDH proteins, with gray value quantification normalized to GAPDH. *P<0.05, **P<0.01, ***P<0.001, ****P<0.0001. Data are expressed as mean ± standard deviation (n=3), data normalization: gray values normalized to the Control group with GAPDH as an internal reference.

Western blot analysis further revealed the molecular mechanism (**Figure 9**). Silencing Rpl23a not only significantly inhibited NF-κB p65 phosphorylation (p-NF-κB p65) but also downregulated the expression of the pro-apoptotic protein Bax while upregulating the anti-apoptotic protein Bcl-2. Overexpression of Rpl23a produced the completely opposite effect: significantly enhancing NF-κB pathway activation while simultaneously upregulating Bax expression and downregulating Bcl-2 expression. Total NF-κB p65 protein levels showed no significant differences among groups. These results indicate that Rpl23a may promote nucleus pulposus cell apoptosis by synergistically activating the NF-κB pathway and altering the balance of apoptosis-regulating proteins.

The above *in vitro* results confirm that Rpl23a may participate in intervertebral disc inflammation amplification, matrix degradation, and nucleus pulposus cell damage by regulating the NF-κB pathway, consistent with bioinformatics predictions.

### 3.6 Regulatory effects of swimming on rat behavior and the Rpl23a/NF−κB pathway

#### 3.6.1 Swimming intervention improves general status and pain behaviors in rats

**Figure 10:**
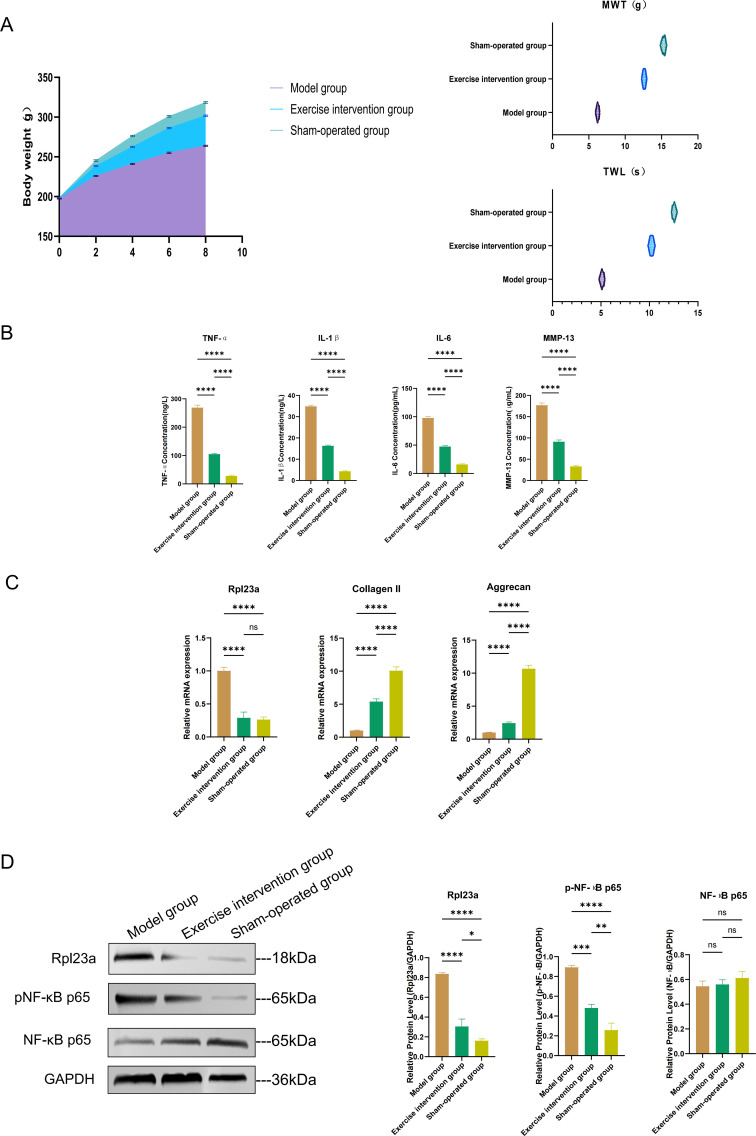
Swimming exercise improves behavior, inflammation, matrix metabolism, and the Rpl23a/NF−κB pathway in IVDD rats. **(A)** Trends in rat body weight over intervention time, mechanical withdrawal threshold (MWT), and thermal withdrawal latency (TWL); **(B)** Protein concentrations of TNF−α, IL−1β, IL−6, and MMP−13 in intervertebral disc tissue measured by ELISA; **(C)** mRNA expression levels of Rpl23a, type II collagen, and aggrecan in intervertebral disc tissue analyzed by RT-qPCR; **(D)** Protein levels and quantitative analysis of Rpl23a, p−NF−κB p65, and total NF−κB p65 detected by Western blot, normalized to GAPDH as an internal reference. SD rats were divided into sham group, model group, and swimming intervention group. Behavioral data: n=10 per group; molecular biological data: n=3 per group. Data are presented as mean ± standard deviation (x¯ ± s). *P<0.05, **P<0.01, ***P<0.001, ****P<0.0001 vs. sham group; P<0.0001 vs. model group. MWT, mechanical withdrawal threshold, unit: g; TWL, thermal withdrawal latency, unit: s.

Body weight monitoring: Compared with the sham group, the body weight growth rate of rats in the model group was significantly slower (P < 0.05). The body weight growth rate was significantly restored in the swimming intervention group compared with the model group (P < 0.05), suggesting that swimming exercise improves the general health and nutritional metabolism of IVDD model rats. (**Figure 10A**).

Pain behavioral results: Compared with the sham group, the mechanical withdrawal threshold (MWT) was significantly decreased and the thermal withdrawal latency (TWL) was markedly shortened in the model group (P < 0.0001), indicating that the IVDD model successfully induced low back pain–related hyperalgesia in rats. After 8 weeks of swimming intervention, MWT was significantly increased and TWL was markedly prolonged in the intervention group (P < 0.0001), demonstrating that swimming effectively alleviates IVDD−induced mechanical hyperalgesia and thermal hyperalgesia, thereby improving low back pain–related clinical symptoms in rats. (**Figure 10A**).

#### 3.6.2 Exercise reduces inflammatory factor levels in disc tissue

ELISA detection (**Figure 10B**) showed that compared to the sham-operated group, protein concentrations of pro-inflammatory factors TNF-α, IL-1β, IL-6, and MMP-13 were significantly elevated in the intervertebral disc tissues of IVDD model group rats (P < 0.0001). After 8 weeks of swimming exercise intervention, the levels of these inflammatory mediators were significantly reduced (P < 0.0001).

#### 3.6.3 Exercise downregulates Rpl23a expression and promotes matrix synthesis

Gene expression analysis (**Figure 10C**) showed that Rpl23a mRNA expression was upregulated in the model group, while Collagen II and Aggrecan mRNA expression was downregulated. Exercise intervention effectively reversed this trend, significantly reducing Rpl23a expression (P < 0.05) and partially restoring the expression levels of matrix synthesis genes.

#### 3.6.4 Exercise inhibits NF-κB pathway activation in disc tissue

Protein level detection results were consistent with gene trends (**Figure 10D**). Rpl23a protein and p-NF-κB p65 protein levels were significantly increased in the model group. In the exercise intervention group, both expressions were significantly inhibited (P < 0.05), while total NF-κB p65 protein levels showed no difference among groups.

*In vivo* results further support that the Rpl23a/NF-κB axis is an important regulatory link in IVDD and a key target for swimming to exert protective effects.

## 4 Discussion

This study identified Rpl23a as a core regulatory molecule related to IVDD pathogenesis through integrated bioinformatics screening. Bulk transcriptome data confirmed that Rpl23a was significantly upregulated in degenerated intervertebral disc specimens, and single-cell sequencing localized its main expression in fibroblasts, suggesting that Rpl23a may participate in IVDD progression by regulating fibroblast-mediated processes. Subsequent experimental verification confirmed that elevated Rpl23a in degenerated tissues drives inflammatory responses and extracellular matrix degradation by activating the NF-κB pathway, further impairing nucleus pulposus cell survival, disrupting apoptosis balance, and accelerating disease progression. The combination of transcriptome localization and functional verification provides new insights into the IVDD molecular regulatory network and identifies independent cell therapeutic targets.

The non-ribosomal functions of ribosomal proteins are important regulatory factors of inflammatory and stress pathways (14). This study found that upregulated Rpl23a in degenerated intervertebral discs was closely related to NF-κB pathway activation and increased pro-inflammatory mediator secretion. Single-cell level further confirmed that Rpl23a was specifically enriched in fibroblasts (a cell type rich in inflammation-related signaling molecules), highlighting the cell type specificity of this regulatory axis. This result is consistent with the classical NF-κB activation mode: upstream signals activate the IKK complex, trigger nuclear translocation, and initiate inflammatory gene transcription (15). Functional experiments showed that Rpl23a silencing inhibited NF-κB activation, while reducing Bax and increasing Bcl-2; overexpression had the opposite effect. These molecular changes corresponded to cell viability changes detected by CCK-8, supporting that Rpl23a regulates apoptosis-related gene expression through an NF-κB-dependent mechanism, ultimately impairing nucleus pulposus cell survival. NF-κB signaling regulates matrix degrading enzymes and apoptosis-related genes in IVDD (4, 16), and this study locates Rpl23a as a potential upstream regulator of this pathway. Rpl23a can regulate nucleus pulposus cell fate, providing a new target for the development of pathway-specific inhibitors. After clarifying the regulatory role of Rpl23a, we further analyzed it in the overall intervertebral disc immune microenvironment to comprehensively understand its pathogenic mechanism.

Disordered cell microenvironment is a key determinant of IVDD development (17). Single-cell sequencing intercellular communication analysis showed that macrophages and fibroblasts formed a dominant communication axis in degenerated intervertebral discs, transmitting two-way signals through TGFβ pathway ligand-receptor pairs. Fibroblasts became a signal integration hub, receiving various inflammation and repair-related signals. This intercellular structure is consistent with the functional characteristics of Rpl23a: its fibroblast-restricted expression localizes it to the main recipient cells of macrophage-derived signals. This study observed active TGFβ signal communication between macrophages and fibroblasts, and Rpl23a was specifically highly expressed in fibroblasts, suggesting a potential functional correlation between the two. Rpl23a participates in this interaction, responds to macrophage-secreted factors (such as TGFβ), and further amplifies the inflammatory cascade through NF-κB activation, forming a pathological cycle: macrophage signals upregulate fibroblast Rpl23a, drive NF-κB-dependent inflammatory mediator release, and ultimately lead to nucleus pulposus cell damage. This theoretical framework improves the current understanding of the IVDD molecular network. Since Rpl23a is a key upstream molecule of the IVDD immune-inflammatory pathway, whether the clinically used exercise rehabilitation therapy may exert protective effects by targeting this molecule has become the focus of our next step.

Exercise intervention has multi-dimensional protective effects on spinal degenerative diseases. This study confirmed that swimming training significantly reduced Rpl23a expression in intervertebral disc tissues of IVDD model rats and inhibited NF-κB pathway activation. Combined with the specific expression pattern of Rpl23a, it is speculated that exercise regulates Rpl23a expression to interrupt the macrophage-fibroblast communication axis and downstream NF-κB signals, and this cascade may be the basis for the anti-inflammatory, matrix-protective, and pro-survival effects of nucleus pulposus cells. Swimming is characterized by low joint load and full-body participation, which can improve intervertebral disc nutrient supply, promote metabolic waste clearance, and regulate local immune homeostasis (10, 18). Importantly, coordinated limb movement and joint activity can strengthen the core and paraspinal muscle groups, improve segmental stability, and reduce intervertebral disc mechanical load (19). The above comprehensive mechanisms jointly regulate the Rpl23a-mediated inflammatory pathway and related intercellular communication networks to maintain intervertebral disc structure and function. After clarifying the intrinsic link between Rpl23a and exercise intervention, this study still has some limitations, which can provide directions for future research.

Recent studies have shown that exercise regulates IVDD through multiple mechanisms such as autophagy regulation, oxidative stress reduction, and cell senescence modification (20–22). Based on the results of this study, future research should explore whether Rpl23a participates in exercise-regulated autophagy or antioxidant defense pathways, and whether exercise can indirectly regulate Rpl23a expression by altering the dynamics of macrophage-fibroblast communication. In addition, comparing the effects of different exercise modes such as endurance, resistance, and flexibility training on Rpl23a expression, cell function, and intervertebral disc health can provide a basis for formulating personalized rehabilitation strategies for IVDD patients.

In addition, this study has the following limitations. First, the nucleus pulposus cells used in *in vitro* experiments differ in pathological status from clinically degenerated cells. Although this study integrated human cells and rat models for verification, and the overall trend was consistent, cross-species and cross-tissue sources may bring potential deviations, and the conclusion needs to be further confirmed in clinical samples. Second, this study adopted a rat acute puncture degeneration model, dominated by rapid inflammation and structural damage, which is significantly different from the pathological characteristics of clinical chronic and progressive degeneration. The long-term effects of Rpl23a and swimming still need to be verified in a model closer to the clinic. Finally, the mechanism discussion only focused on the NF-κB pathway and did not involve other signaling pathways; only the effect of swimming intervention was observed, lacking comparison of different exercise modes; and the regulatory effect of Rpl23a on intercellular communication was not analyzed in a co-culture system, and the relevant mechanism needs to be further improved.

## 5 Conclusion

This study suggests that Rpl23a is specifically highly expressed in degenerated intervertebral discs and may aggravate intervertebral disc inflammation and matrix degradation by regulating the NF-κB pathway, which is closely related to the process of IVDD. Swimming can downregulate Rpl23a expression, inhibit excessive activation of the NF-κB pathway, and improve the disordered immune microenvironment of intervertebral discs, thereby delaying the degeneration process to a certain extent. Therefore, this study preliminarily reveals that Rpl23a may be a potential key molecule regulating intervertebral disc immune inflammation and a potential target for exercise to exert intervertebral disc protective effects, providing a preliminary theoretical reference and experimental clues for molecular targeted therapy and precise exercise rehabilitation of IVDD.

## Data Availability

The original contributions presented in the study are included in the article/supplementary material. Further inquiries can be directed to the corresponding author.
